# LEM domain containing 1 promotes pancreatic cancer growth and metastasis by p53 and mTORC1 signaling pathway

**DOI:** 10.1080/21655979.2022.2047404

**Published:** 2022-03-14

**Authors:** Xiang Cao, Na Yao, Zidan Zhao, Yue Fu, Yuting Hu, Ping Zhu, Weihai Shi, Liming Tang

**Affiliations:** a Department of General Surgery, The Affiliated Changzhou No. 2 People’s Hospital of Nanjing Medical University, Changzhou, Jiangsu, China; b Department of Thyroid & Breast Surgery, The Affiliated Wuxi Hospital of Nanjing University of Traditional Chinese Medicine, Wuxi City Hospital of Traditional Chinese Medicine, Wuxi, Jiangsu, China

**Keywords:** Pancreatic cancer, LEMD1, cancer aggressiveness, p53, mTORC1

## Abstract

Pancreatic cancer (PC) is a common type of malignancy originating from the epithelium of the pancreatic duct, with the most lethal feature and worst prognosis. LEM domain containing 1 (LEMD1) is overexpressed in multiple tumor tissues and plays a key role in cancer carcinogenesis and progression. However, little is known about the potential of LEMD1 in PC. In this study, we explored the clinical values, as well as the potential roles and mechanisms of LEMD1 in PC. We, for the first time, showed that LEMD1 was upregulated in PC and negatively correlated with the overall and disease-free survival of patients with PC. Of the function, LEMD1 knockdown inhibited cancer cell growth, migration and invasion, while LEMD1 overexpression promoted tumor aggressiveness. The tumor-promoting influences of LEMD1 in PC were also proved by in vivo assays. Mechanistically, GSEA identified that LEMD1 promoted PC aggressiveness, as well as affecting cell cycle dysregulation and apoptosis resistance, by p53 suppression and the activation of the mTORC1 signal pathway. In short, LEMD1 could serve as a valuable prognostic candidate and a potential therapeutic target of PC.

**Abbreviations**: ATCC: American Type Culture Collection; CCK-8: Cell counting kit 8; CDK: Cyclin-dependent kinases; CTA: Cancer-testis antigen; DMEM: Dulbecco’s Modified Eagle’s Medium; ECL: enhanced chemiluminescence; FBS: Fetal bovine serum; GEO: Gene Expression Omnibus; LEMD1: LEM domain containing 1; mTOR: mammalian target of rapamycin; NC: Negative control; PC: Pancreatic cancer; PVDF: Polyvinylidene difluoride membranes; qRT-PCR: Quantitative real-time polymerase chain reaction; SDS-PAGE: Sodium dodecyl sulfate polyacrylamide gel electrophoresis; SD: Standard deviation; SKP2: S-Phase kinase-associated protein 2; TAA: Tumor-associated antigen; TBST: Tris-buffered Saline Tween-20; TCGA: The Cancer Genome Atlas.

## Graphical abstract

**Figure:**
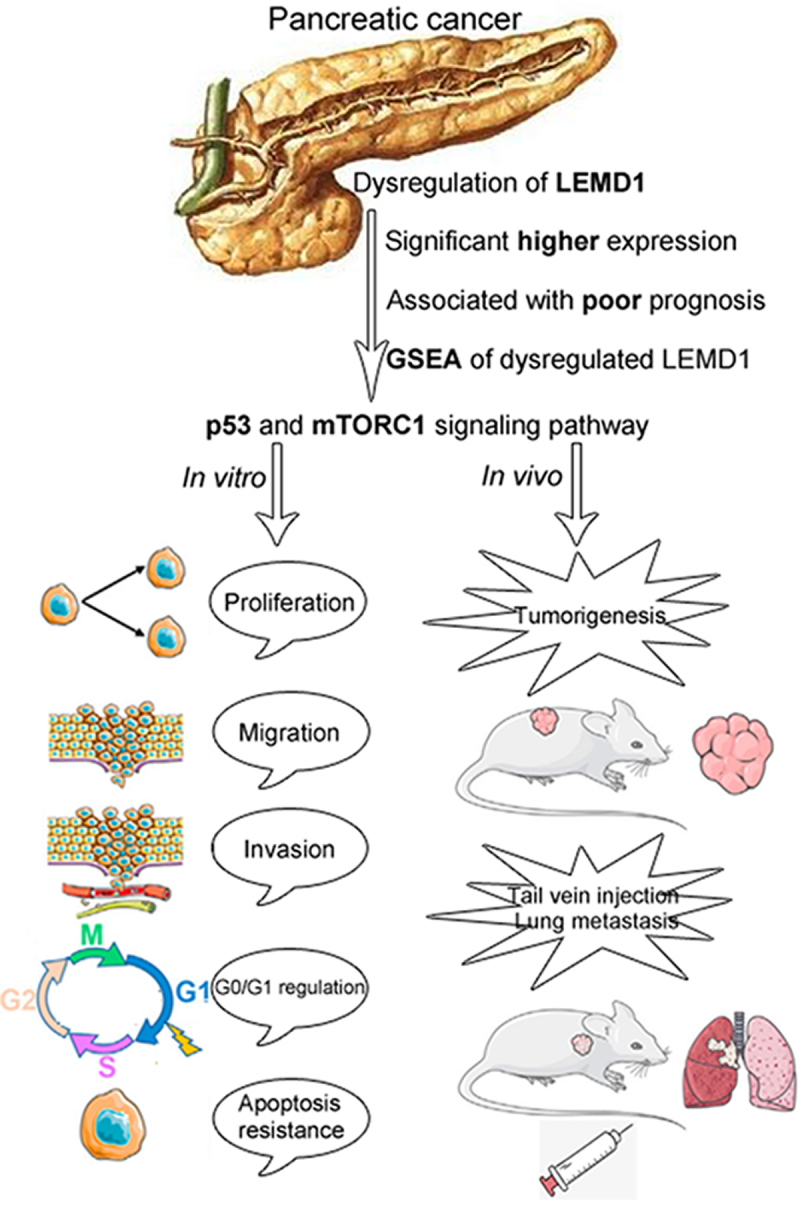

## Introduction

Pancreatic cancer (PC) is one of the most aggressive malignancies, with an average 5-year survival rate of less than 8% and a median overall survival of 6 months [1]. As reported, more than 80% of patients with PC lacking nonspecific symptoms, such as bloating and abdominal pain, are frequently diagnosed at locally advanced stage or with even extra-pancreatic metastasis [2]. Surgical resection and adjuvant chemotherapy are considered the major therapeutic strategies for PC in clinic. Unfortunately, early lymph node metastasis, low rate of radical resection (less than 20%) and high chemoresistance (either ineffective or effective only for a short duration) are all critical reasons for the poor prognosis of PC [3]. In this regard, it is urgently important to explore mechanisms of PC carcinogenesis and to seek novel therapeutic biomarkers.

LEM domain containing 1 (LEMD1, also reported as *CT50* and *LEMP-1*) is identified to be a novel candidate gene of the cancer-testis antigens (CTAs) family with several features of ideal biotargets for cancer immunotherapy and early detection due to the characteristics that are normally present in the testis and aberrantly expressed in multiple cancer cells [4,5]. Upregulated CTAs expression has been correlated with advanced disease and worse survival. For LEMD1, with the update of studies, its overexpression has been proved in a series of malignancies, such as prostate cancer, oral squamous cell carcinoma, gastric cancer and colorectal cancer [^5–8^]. Of importance, multiple tumor aggressiveness has been attributed to the dysregulation of LEMD1 in certain cancer, for instance, including cancer cell proliferation, cell cycle, cell apoptosis, metastasis and endothelial transmigration [6,7]. However, limited research has reported the expression profile, potential role and molecular mechanism of LEMD1 in PC.

Herein, in an effort to find key oncogenes, we first explored the underlying mechanism of LEMD1 in the progression of PC. Our data first elucidated that LEMD1 was significantly upregulated in PC, and patients with a high LEMD1 level were more likely to suffer poor prognosis. Further studies showed that LEMD1 promoted the progression of PC, as well as influenced the cell cycle and apoptosis resistance, by the suppression of p53 and activation of mTORC1 (mammalian target of rapamycin) signaling pathway. All findings supported the oncogenic roles in cancer cell aggressiveness, highlighting its potential for PC prognosis and therapy.

## Materials and methods

### Bioinformatics analysis

Expression comparisons of tumor and normal samples of multiple malignancies from GTEx (Genotype-Tissue Expression) and TCGA (The Cancer Genome Atlas) projects were carried out by applying GEPIA 2 (Gene Expression Profiling Interactive Analysis 2) (http://gepia2.cancer-pku.cn/) standard processing pipeline [9]. In detail, the expression profile of LEMD1 and survival information of PC patients (n = 179) were downloaded from TCGA. A total of 163 patients were selected for Kaplan-Meier plot analysis, and others were excluded due to lack of survival information (Overall survival (OS) & Disease-free survival (DFS)). The cutoff value for higher and lower levels was set as the median value of gene expression. A series of PC related chip-seq data sets including GSE15471, GSE62452 and GSE62165 downloaded from Gene Expression Omnibus (GEO) were applied to determine the expression differences of LEMD1 in PC samples.

Furthermore, we selected and applied GSE15471- and GSE62452-containing expression data of PC samples for gene set enrichment analysis (GSEA), which was successfully carried out using the GSEA v3.0 tool (http://www.broadinstitute.org/gsea). In detail, we divided all PC samples in each data set into high and low expression groups according to the median level of LEMD1. The P value of normalized enrichment score (NES) < 0.05 and false discovery rate (FDR) < 0.25 were considered to have statistical significance. For GSEA, we assessed the underlying mechanisms of LEMD1 in cancer aggressiveness using the hallmarks of cancer-related gene signatures.

### Cell culture and transfection

All human PC cell lines (Colo357, BxPC-3, MIA PaCa-2, CFPAC-1, PANC-1 and SW1990) obtained from the Shanghai Cell Bank were cultured in a humid atmosphere of 5% CO_2_ with DMEM (Dulbecco’s Modified Eagle’s Medium) supplemented with FBS (fetal bovine serum; 10%) and penicillin/streptomycin (1%) (Gibco, USA). HPNE, known as the normal pancreatic ductal cell line, was purchased from American Type Culture Collection (ATCC; USA) and incubated according to the protocols. Rapamycin, the inhibitor of mTORC1 signaling, was purchased from the Cell Signaling Technology Biotech (CST, Danvers, MA, USA).

The biologically specific short hairpin RNAs (shRNAs) targeting human LEMD1 and its negative control (shNC) were synthesized by GenePharma Biotech (Shanghai, China) and inserted into pLKO.1 vector. LEMD1-expressing plasmid (pcDNA-LEMD1) and its control empty vector were designed by RiboBio Biotech (Guangzhou, China) and inserted into pGLV3/H1/EGFP vector. To generate target lentivirus for LEMD1 down- and upregulation, Lipofectamine 3000 (Invitrogen) was applied to cotransfect the packaging vectors and lentiviral vectors into 293 T cells according to the manufacturer’s instructions. The stably transfected PC cells were selected by Puromycin appropriately.

### Quantitative real-time polymerase chain reaction (qRT-PCR)

Total RNA isolated from PC cells and samples using TRIzol reagent (Invitrogen) was reversely transcribed into cDNA using TaKaRa’s PrimeScript RT (reverse transcription) reagent (RR036A). The mRNA level status of designated genes was calculated by qRT-PCR carried out using an ABI 7900HT Real-Time PCR system (Applied Biosystems, USA) with SYBR Green Master Mix (TaKaRa) according to the manufacturers’ specifications. Fold differences were normalized to β-actin according to the formula 2^−ΔΔCt^ (Ct means the cycle threshold). The sequences of primers were shown as follows:

LEMD1 forward 5ʹ‐ACTTCTATCATCATGGTGGATG‐3ʹ;

LEMD1 reverse 5ʹ‐GATCTGTGAGAGCAGCACAG‐3ʹ;

β-actin forward 5ʹ‐GCATCGTCACCAACTGGGAC‐3ʹ;

β-actin reverse 5ʹ‐ACCTGGCCGTCAGGCAGCTC‐3ʹ.

### Western blot

Total protein was prepared from PC cells or tissue samples by using RIPA buffer with the phenylmethylsulfonyl fluoride (PMSF), phosphatase, and protease inhibitors. Equal amounts of protein (10–35 μL) were run on sodium dodecyl sulfate‐polyacrylamide gel electrophoresis (10% SDS-PAGE), transferred to a polyvinylidene difluoride (PVDF) membrane, blocked in a mixture of Tris‐buffered saline with Tween 20 (TBST) with 5% nonfat milk and next incubated overnight at 4°C with primary antibodies as follows: LEMD1 (Catalog #ab201206; 1:1000; Abcam, UK), CDK2 (Catalog #2546; 1:1000; CST; USA), CDK4 (Catalog #12,790; 1:1000; CST; USA), p21 (Catalog #2947; 1:1000; CST; USA), p27 (Catalog #3686; 1:1000; CST; USA), SKP2 (Catalog #2652; 1:1000; CST; USA), p53 (Catalog #2524; 1:1000; CST; USA), p53-pSer15 (Catalog #9284; 1:1000; CST; USA), p53-pSer46 (Catalog #2521; 1:1000; CST; USA), Bax (Catalog #ab32503; 1:1000; Abcam; UK), Bcl-2 (Catalog #ab196495; 1:800; Abcam; UK), mTOR (Catalog #2972; 1:1000; CST; USA), p-mTOR-Ser2448 (Catalog #5536; 1:1000; CST; USA), P70S6K (Catalog #2708; 1:1000; CST; USA), p-P70S6K (Thr389) (Catalog #9234; 1:1000; CST; USA), S6 (Catalog #14467; 1:1000; CST; USA) and p-S6-Ser240/244 (Thr389) (Catalog #9468; 1:1000; CST; USA). The membranes were incubated with HRP-conjugated antimouse or antirabbit IgG at room temperature for 2 h and washed 3 times with TBST. β-Actin was used as an internal control. All protein signals were calculated using a Chemiluminescence HRP Substrate (Millipore) and Enhanced Chemiluminescence (ECL) system.

## Cell proliferation assays

### Cell counting kit 8 (CCK-8)

Cells (2 × 10^3^ per well) were seeded and grown in 96-well plates for 0, 24, 48, 72 and 96 h before which complete medium (90 μL) and CCK-8 (10 μL; Dojindo, Japan) were added per well. The plates were incubated in the dark for 3 h at 37°C and analyzed at 450 nm absorbance.

### Clone formation

Approximately 6 × 10^2^ PC cells were seeded into a 6-well plate and maintained with the complete medium for a 14-day period, after which crystal violet was used for cell staining prior to quantification of the number of clones.

### Cell migration and invasion

To explore PC cell migration and invasion, Transwell and BioCoat Matrigel chambers (BD Biosciences) were applied for research as described previously [10]. In short, after incubation in serum-free medium for 24 h, cells were seeded into the upper chamber (2.5 × 10^4/^well) containing serum-free medium (200 μL) with an uncoated or Matrigel-coated membrane. Complete medium (700 μL) was added to the lower chamber. After incubation for 48 h at 37°C in a humidified 5% CO_2_ incubator, cells on the upper surface of filters were removed with cotton swab. Following staining with 0.5% crystal violet, cells in five random areas under 100 × magnification were counted, and representative images were photographed under a phase-contrast microscope (Nikon). All assays were repeated in triplicate.

### Cell apoptosis and cell cycle

Cell apoptosis and cell cycle analysis of each transfected cell were assessed using the flow cytometric (FCM) method. For apoptosis detection, cells were harvested in a 6-well plate (80%–90%), resuspended in Annexin V (400 μL), stained with 7-aminoactinomycin (7-AAD) viability solution and phycoerythrin (PE) Annexin V (5 μL) in the dark at room temperature for 20 min and detected using a FACScan flow cytometer (BD, Franklin Lakes, New Jersey). For cell cycle analysis, cells in each group were, respectively, harvested in a 6-well plate, fixed in 75% ethanol overnight at −20°C, washed twice with PBS, stained with PI/RNAse buffer and assessed using a FACScan flow cytometer.

### Animal

All female BALB/c mice aged 4 weeks were purchased from the Model Animal Research Center of Nanjing. All animal assays were approved by the Institutional Animal Care and Use Committee of Nanjing Medical University. For the tumorigenicity model, a total of 100 μL transfected cells (MIA PaCa-2, 1.2 × 10^6^/100 μL; PANC-1, 1 × 10^6^/100 μL) were subcutaneously injected into a single back of nude mice (totally 10 mice for each group). The tumor diameter was calculated every 4 days, and the tumor volume was monitored based on the formula: 0.44 × D^2^ × d (D, latitudinal diameter; d, longitudinal diameter). To investigate the roles of LEMD1 in PC metastasis, fresh cancer cells (MIA PaCa-2, 6 × 10^6^/100 μL; PANC-1, 5 × 10^6^/100 μL) were all carefully injected into the tail vein of mice under anesthesia (a total of 10 mice for each group). An IVIS Lumina II system (Caliper Life Sciences, USA) was used to determine the tumor metastatic nodules weekly. All nude mice used in this study were humanly sacrificed.

### Statistical analysis

A series of public data sets, including GEPIA, TCGA and GEO, were downloaded and used to determine the dysregulation of LEMD1 in PC samples using R (http://www.r-project.org/, version 2.1.0) and Bioconductor [11]. Paired/independent student’s *t*-test and Analysis of Variance (ANOVA) were used to assess comparisons between two groups appropriately. Survival analysis was perfomed by the Kaplan-Meier and log-rank test. All statistical analysis and histograms were completed by applying the SPSS 17.0 software (SPSS Inc.) and the GraphPad Prism software (version 5.01). All differences with p < 0.05 were considered to have statistical significance. All data were expressed as mean ± standard deviation (SD).

## Results

### LEMD1 is upregulated in human PC tissues and cell lines

**Figure 1.**
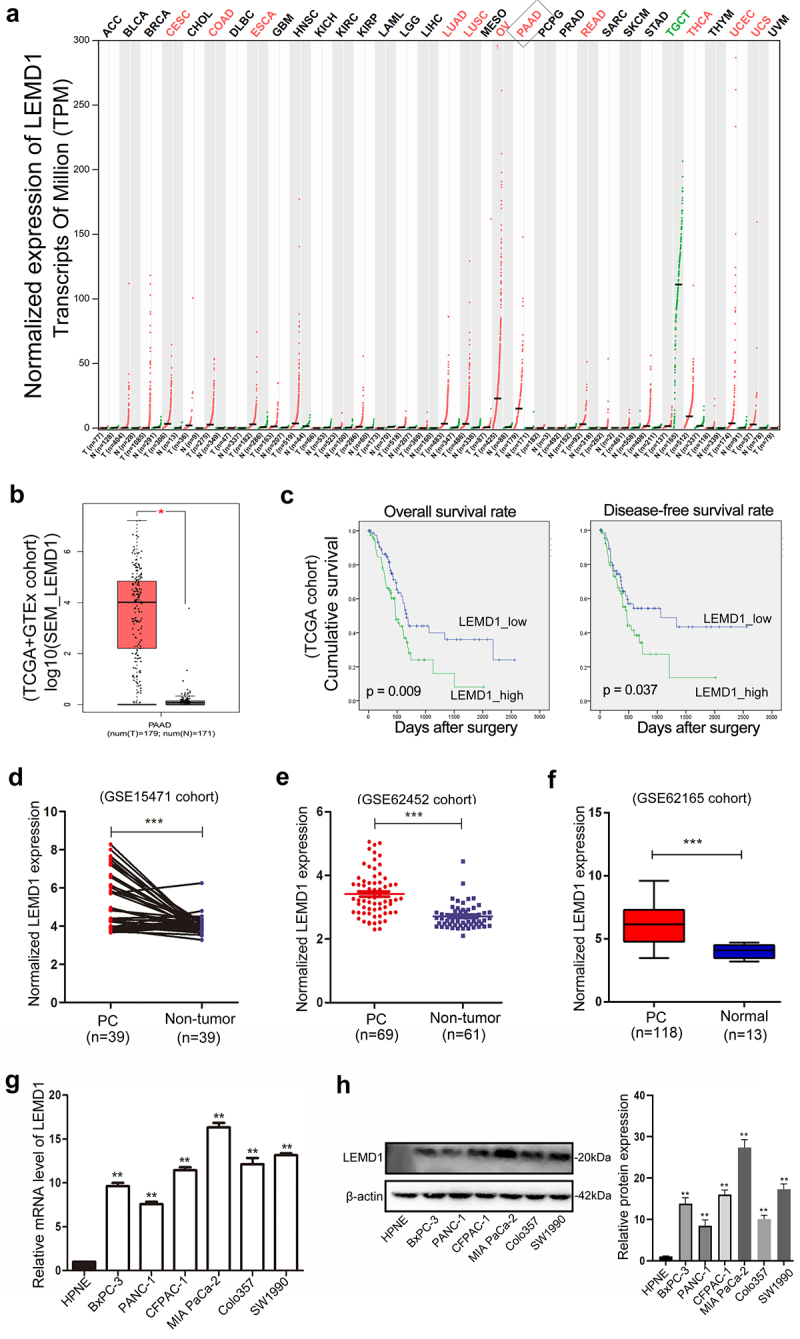
LEMD1 is overexpressed in human PC samples and cell lines.

According to public data from GEPIA, LEMD1 was frequently upregulated in multiple kinds of malignant tumors, such as OV (ovarian serous cystadenocarcinoma), CESC (cervical squamous cell carcinoma) and THCA (thyroid carcinoma) (Figure 1a). In detail, LEMD1 was identified to be significantly overexpressed in PC samples (Figure 1b). Through downloading gene expression and corresponding clinical data of patients with PC from TCGA, a total of 163 patients were included for survival assessments. Of importance, patients with a higher level of LEMD1 indicated a shorter disease-free and overall survival rate (Figure 1c). Moreover, evidence from several specific PC-related GEO data sets (GSE15471, GSE62452 and GSE62165) all elucidated that LEMD1 was significantly upregulated in PC tissue samples relative to the adjacent noncancerous or normal tissue samples (Figure 1d-f). In addition, PC cell lines revealed higher levels of LEMD1 compared with normal pancreatic duct cells (HPNE) (Figure 1g,h). In short, all findings suggested that upregulated LEMD1 may function as a potential prognostic biomarker for patients with PC.

**a** According to the GEPIA, upregulation of LEMD1 commonly exists in multiple kinds of human cancers. **b** Expression profiles of LEMD1 in PC and adjacent normal samples are shown (TCGA and GTEx). **c** Increased level of LEMD1 was negatively associated with overall survival and disease-free survival of patients with PC. Totally, 163 patients were divided based on the median value of LEMD1 expression (TCGA). **d-f** Analysis of the LEMD1 level in human PC tissues and adjacent noncancerous/normal tissues in a series of GEO data sets (**d**: GSE15471; **e**: GSE62452 and **f**: GSE62165). **g and h** LEMD1 mRNA and protein expression in the human pancreatic ductal epithelial cell line (HPNE) and PC cell lines was evaluated by qRT-PCR and western blot. * *p* < 0.05, ** *p* < 0.01, *** *p* < 0.001. All experiments were repeated at least three times.

### Knockdown of LEMD1 inhibits PC cell proliferation, migration and invasion

**Figure 2.**
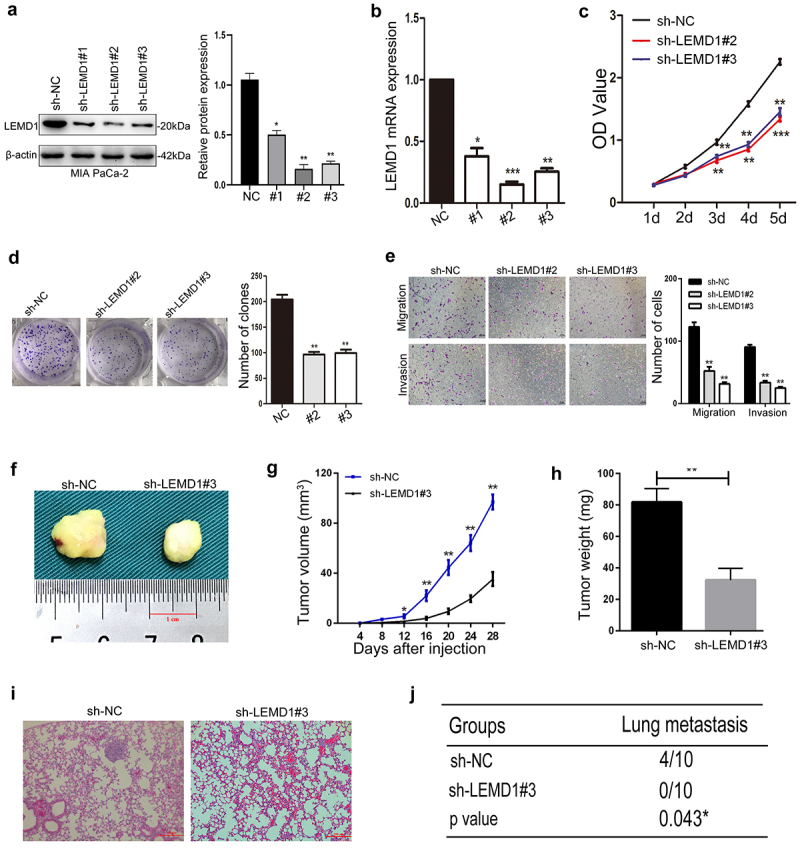
Knockdown of LEMD1 inhibits cancer cell growth, migration and invasion in PC.

To explore the aggressive roles of LEMD1 in PC, LEMD1 was effectively silenced by specific short hairpin RNAs (shRNAs) in MIA PaCa-2, which showed a highest level of LEMD1 (Figure 1g,h). As detailed, the interference efficiencies of shLEMD1s were validated (Figure 2a,b). For above shLEMD1s, shLEMD1#2 and shLEMD1#3 could remarkedly inhibit the production of LEMD1 and were selected for further functional assays.

**a and b** Western blot and qRT-PCR analysis of LEMD1 mRNA and protein expression in MIA PaCa-2 cells treated with sh-LEMD1s.**c** Growth rates of sh-LEMD1s and sh-NC-transfected PC cells were explored by CCK-8. **d** Effects of LEMD1 silencing on clone formation. **e** Comparisons of migration and invasion by counting the number of cells that invaded through the transwell without or with Matrigel (Magnification: 100 ×). **f** Representative figure of subcutaneous tumors in LEMD1 knockdown and its negative control group. **g** Tumor volume was measured at the indicated day. **h** Mean tumor weight. **i** H-E images of the tail vein injecting model indicates lung metastatic nodules formed in each group (n = 10) (Magnification: 100 ×). **j** Number of mice with metastatic foci in the lung was measured, as shown in the table. * *p* < 0.05, ** *p* < 0.01, *** *p* < 0.001. All assays were repeated at least three times.

Simultaneously, CCK-8 and cell clone experiments were used to study the influences of LEMD1 on PC cell growth. The results of CCK-8 indicated that the growth rates of shLEMD1#2- and shLEMD1#3-transfected cancer cells were significantly suppressed compared to those of shNC-transfected cells (Figure 2c). The number of cell clones was also markedly inhibited in PC cells with LEMD1 knockdown (Figure 2d). Next, transwell was used to detect cell metastasis. The number of migrated and invaded PC cells was significantly decreased in the LEMD1 knockdown group relative to its negative control (Figure 2e).

To extend *in vivo* observations, the subcutaneous xenograft model and tail vein metastasis nude mice model were further established to investigate the influences of LEMD1 on PC cell tumorigenicity and metastasis. As shown, the growth rate, weight and volume of subcutaneous xenografts were evidently decreased in LEMD1-silencing groups relative to their negative control (Figure 2f-h). Furthermore, findings of the tail vein model revealed that no lung colonizations formed in MIA PaCa-2 cells with the decreasing level of LEMD1, whereas 4 nude mice of the negative control group presented lung metastatic colonizations (Figure 2i,j). Taken together, all observations supported that LEMD1 knockdown could inhibit PC cell growth and metastasis both *in vitro* and *in vivo*.

### Overexpression of LEMD1 promotes PC proliferation, migration and invasion

**Figure 3.**
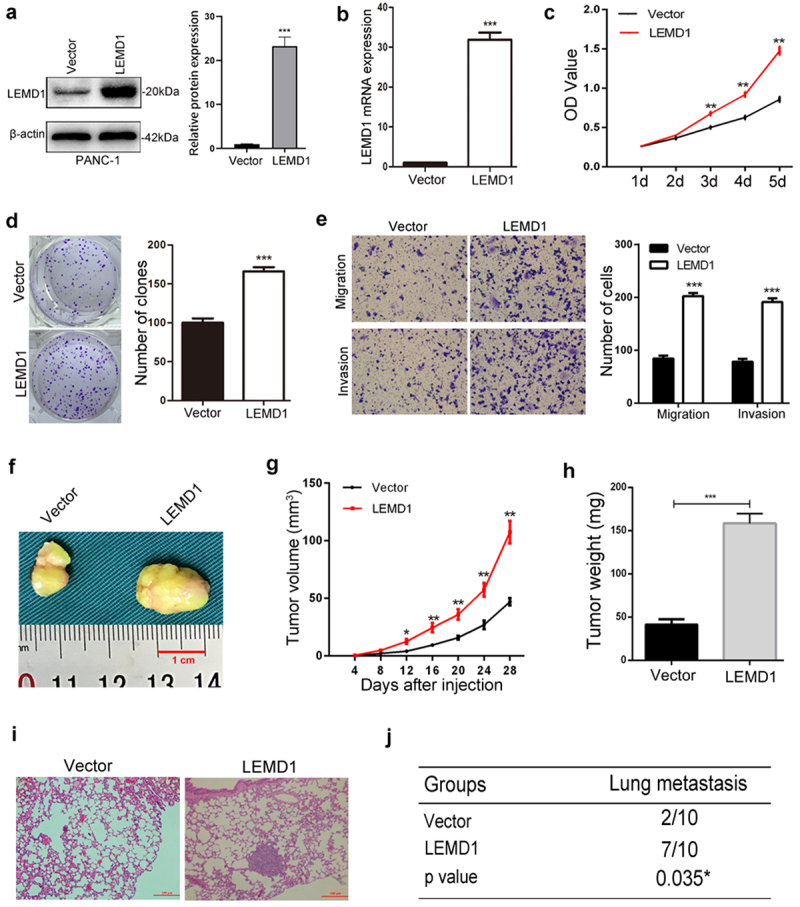
LEMD1 facilitates PC cell growth, migration and invasion.

To explore the effects of LEMD1 in PC aggressiveness, pcDNA-LEMD1 lentivirus was used to upregulate the LEMD1 level in PANC-1, which showed a lowest level in PC cell lines (Figure 1g,h). The overexpression of LEMD1 was further validated by qRT-PCR and western blot (Figure 3a,b).

**a and b** Efficiencies of LEMD1 overexpression in PANC-1 cells were proved by western blot and qRT-PCR.**c and d** CCK-8 and clone formation assays were applied to assess the cell growth rate in PC cells with ectopic LEMD1. **e** Influences of overexpressed LEMD1 on cell migration and invasion were detected by transwell as indicated (Magnification: 100 ×). **f** Representative image of subcutaneous xenograft tumor is shown. **g** Tumor volume was calculated at the indicated time. **h** Tumor weight was measured. **i** H-E staining showing lung colonizations in LEMD1 overexpression and the control group (n = 10) (Magnification: 100 ×). **j** Number of mice with lung metastatic foci was calculated, as depicted in the table. * *p* < 0.05, ** *p* < 0.01, *** *p* < 0.001. All assays were repeated at least three times.

The CCK-8 and clone formation assay revealed that LEMD1 upregulation significantly enhanced the proliferation of PANC-1 cells (Figure 3c,d). Meanwhile, the Transwell assay showed that overexpression of LEMD1 remarkedly increased PC cell migration and invasion (Figure 3e). For *in vivo* explorations, the growth rate, weight and volume of subcutaneous tumors generated from LEMD1 overexpression cells were obviously larger and heavier than those of the NC group (Figure 3f-h). In addition, by using the tail vein injecting model, increased lung metastatic colonizations were formed in PANC-1 cells with a higher level of LEMD1, while totally 7 mice (10 mice each group) presented lung metastasis that is proved by the histologic examinations (Figure 3i,j). All findings supported the promotive roles of LEMD1 in PC proliferation and metastasis.

### LEMD1 influences G0/G1 phase transition and inhibits PC apoptosis

Although we found that LEMD1 could promote cancer cell proliferation, the underlying mechanisms remained still unknown. Since the cell cycle and apoptosis rate were generally reported to be associated with the activity of cell proliferation, we next focused on the role of LEMD1 in the PC cell cycle and apoptosis.

**Figure 4.**
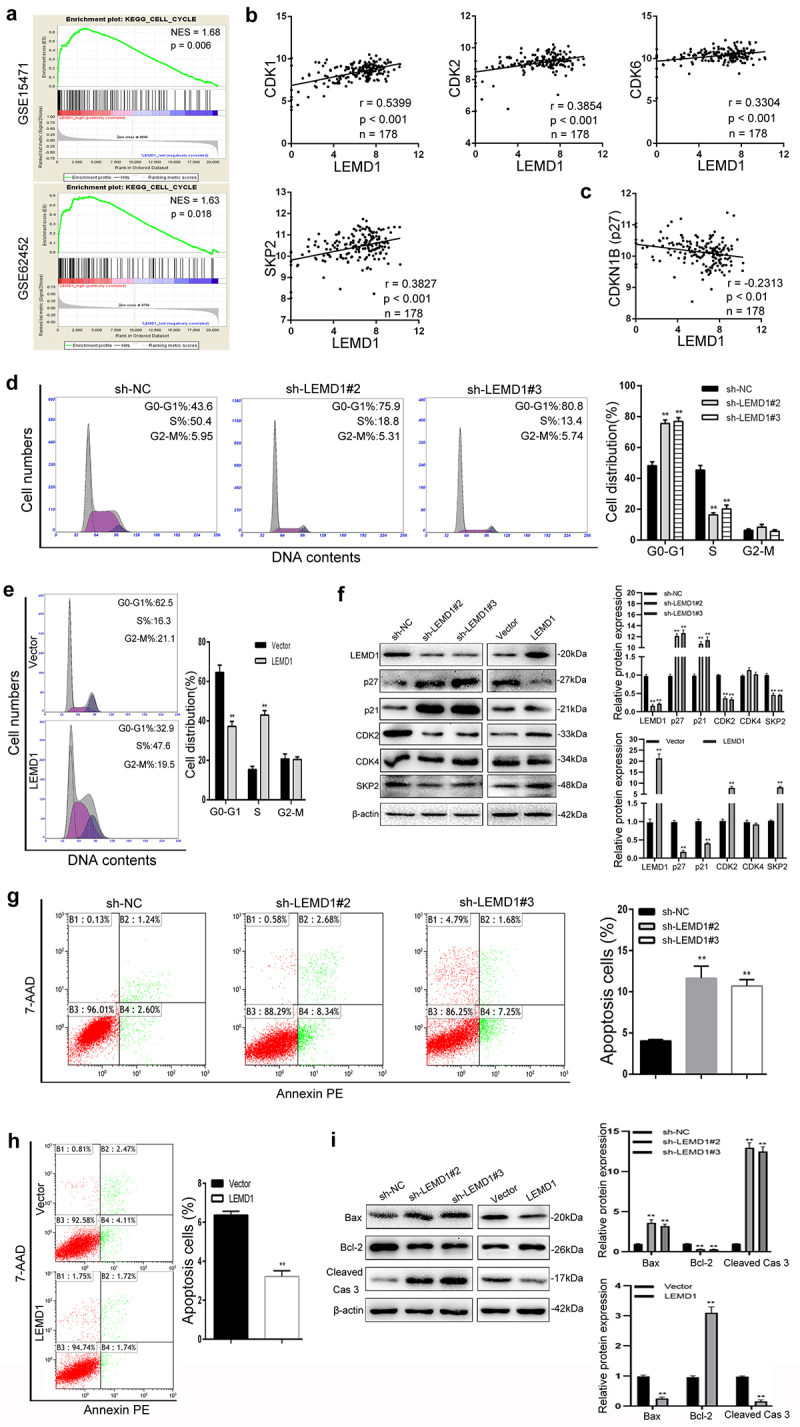
LEMD1 influences G0/G1 transition and inhibits PC apoptosis.

Of interest, by using a powerful tool to analyze the gene expression data, GSEA of two PC-related data sets (GSE15471 & GSE62452) suggested that LEMD1 expression was significantly associated with the cell cycle (NES = 1.68, p = 0.006; NES = 1.63, p = 0.018) (Figure 4a). Additionally, correlation analysis of the data from TCGA (n = 178) showed that LEMD1 expression was positively associated with several cell cycle-related genes, including *CDK1, CDK2, CDK6* and *SKP2*, in PC samples (Figure 4b). Meanwhile, a significant negative relationship between the level of LEMD1 and CDKN1B (p27) was identified in PC samples (Figure 4c). All bioinformatics indicated the potential between LEMD1 and the cell cycle in PC cells. Subsequently, we next conducted the flow cytometry analysis. The results showed that LEMD1 knockdown increased the percentage of cells in the G0/G1 phase and resulted in cell cycle arrest at G0/G1 in PC (Figure 4d). However, overexpression of LEDM1 presented a converse trend (Figure 4e). To investigate how silencing of LEMD1 leads to G0/G1 arrest, the levels of multiple proteins that play vital roles in the G0/G1 phase were calculated by western blot. As depicted, the productions of CDK2 and SKP2 were positively regulated by LEMD1 in cancer cells (Figure 4f). P21 and p27, reported as essential regulators of the cell cycle at G0/G1, were significantly downregulated in PC cells with ectopic LEMD1 (Figure 4f). Conversely, knockdown of LEMD1 promoted p21 and p27 levels (Figure 4f). These data indicated that silencing of LEMD1 could induce cell arrest at G0/G1 by p21 and p27 productions.

**a** GSEA indicated the close relationship between LEMD1 expression and the cell cycle in PC (GSE15471 & GSE62452). **b** LEMD1 expression was positively associated with several cell cycle-related proteins (CDK1, CDK2, CDK6 and SKP2) in PC specimens (TCGA). **c** A negative relationship between the expression of LEMD1 and CDKN1B (p27) was identified in PC samples (TCGA). **d** Flow cytometry analysis of the cell cycle in cancer cells with LEMD1 knockdown. **e** Effects of ectopic LEMD1 on the cell cycle. **f** Western blot for LEMD1, p27, p21, CDK2, CDK4 and SKP2 in LEMD1-altered PC cells as indicated. **g and h** Flow cytometry used to explore the roles of LEMD1 in PC cell apoptosis. **i** Western blot for Bax, Bcl-2 and Cleaved caspase 3 in PC cells as indicated. ** *p* < 0.01. All assays were repeated at least three times.

Furthermore, LEMD1 has been shown to induce apoptosis in PC. As depicted, LEMD1 knockdown significantly increased the percentage of apoptotic cancer cells (Figure 4g). In contrast, overexpression of LEMD1 effectively suppressed cell apoptosis (Figure 4h). The abundant accumulation of cleaved caspase 3 generally indicates cell apoptosis progression. Thus, we next detected several apoptosis-related proteins, such as cleaved caspase 3, bax and bcl-2. As expected, the protein expression of cleaved caspase 3 was negatively influenced in PC cells with LEMD1 down- or upregulation (Figure 4i). In addition, depletion of LEMD1 remarkedly increased bax expression and decreased the level of bcl-2 (Figure 4i). Conversely, ectopic LEMD1 presented an opposite trend in PC cells (Figure 4i).

Taken together, evidence in this aspect supports that LEMD1 may promote the growth of PC by facilitating G0/G1 cell cycle transition, as well as apoptosis suppression.

### Effects of LEMD1 on the p53 signaling pathway in PC cells

**Figure 5.**
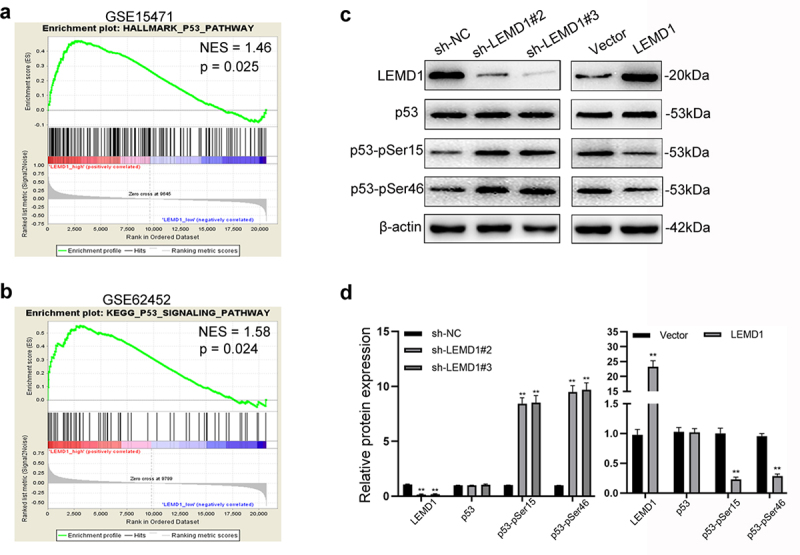
Effects of LEMD1 on the p53 signaling pathway in PC cells.

**Figure 6.**
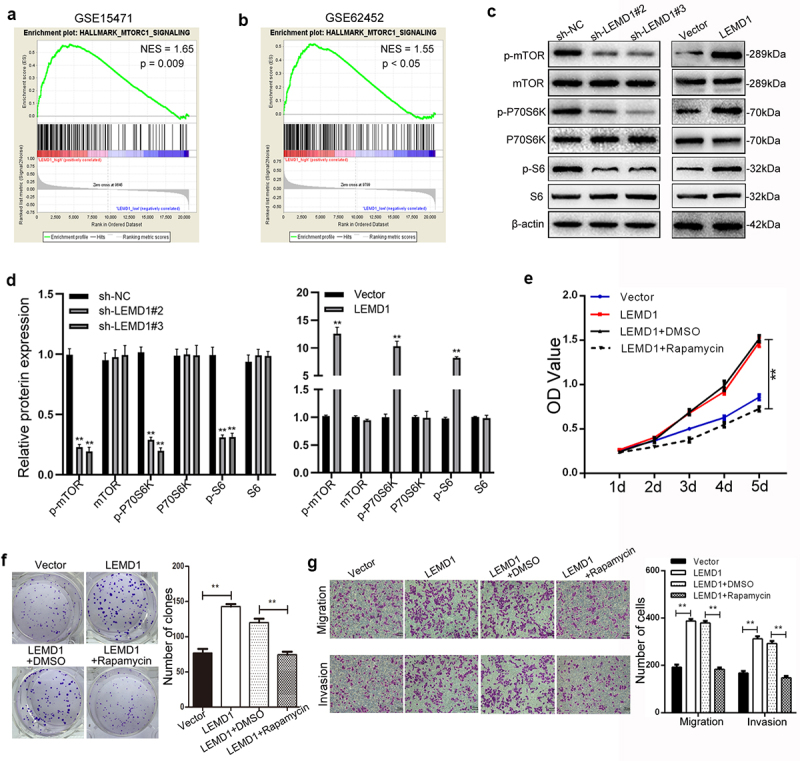
LEMD1 promotes cancer progression by activation of mTORC1 signaling in PC.

Of importance, evidence of GSEA supported that LEMD1 expression was significantly related to p53 signaling (NES = 1.46, p = 0.025; NES = 1.68, p = 0.024) (Figure 5a,b). Thus, we postulated that LEMD1 was most likely to promote cancer aggressiveness via regulating p53 signaling-related apoptosis and cell cycle. As shown, knockdown of LEMD1 significantly stimulated, whereas overexpression of LEMD1 suppressed the phosphorylation and activation of p53 signaling at p53-pSer15 and p53-pSer46 in PC cells (Figure 6c,d). All findings suggested that LEMD1 inhibited p53 signaling in PC.

**a and b** GSEA indicated that LEMD1 expression was closely related to p53 signaling in PC samples (GSE15471 and GSE62452). **c and d** Western blot was applied to prove the potential of LEMD1 in the p53 signaling pathway (p53, p53-pSer15 and p53-pSer46). ** *p* < 0.01. All experiments were repeated at least three times.

**a and b** GSEA of GSE15471 and GSE62452 suggested that LEMD1 levels were significantly related to the mTORC1 signaling pathway in PC. **c and d** Western blot analysis showing the total and phosphorylated levels of mTOR, P70S6K and S6 in LEMD1-altered PC cells. **e and f** CCK-8 and clone formation experiments were used to assess cancer cell proliferation following ectopic LEMD1 or combined with rapamycin treatment. The DMSO was used as the internal control. **g** Cell migration and invasion assessed using transwell in LEMD1-overexpressed cells after treatment of rapamycin or DMSO as indicated (Magnification: 100 ×). ** *p* < 0.01, *** *p* < 0.001. All assays were repeated at least three times.

### LEMD1 stimulates the mTORC1 signaling pathway in PC

A series of signaling pathways, including Wnt, mTORC1, MAPK/ERK and PI3K/AKT, have been increasingly identified to be essential regulators that participate in cancer carcinogenesis [^12–14^]. Of interest, hallmark of GSEA also indicated that LEDM1 was significantly associated with the p53 signaling pathway in PC (NES = 1.65, p = 0.009; NES = 1.55, p < 0.005) (Figure 6a,b). Due to GSEA, we supposed that LEMD1 could activate the mTORC1 signaling pathway, thus facilitating PC cell aggressiveness.

Subsequently, we detected several effectors of the mTORC1 signaling (mTOR, p70 S6 kinase (P70S6K) and S6) by western blot. The phosphorylated level of mTOR, P70S6K and S6 was obviously enhanced in PC cells with LEMD1 knockdown, while decreased in LEMD1-overexpressed cells than the control (Figure 6c,d). However, no significant differences were identified for the total level of mTOR, P70S6K and S6 in LEMD1-altered PC cells (Figure 6c,d). Furthermore, rapamycin, a specific inhibitor of mTORC1, was selected and used to prove the promotive role of LEMD1 in mTORC1 signaling by using a series of rescue functional assays. As presented, ectopic LEMD1 increased the proliferative rate of cancer cells, which was alleviated by rapamycin (Figure 6e). The number of cell clones was enhanced in the LEMD1-upregulated group; however, the mTOR inhibitor dramatically repressed (Figure 6f). Meanwhile, rapamycin also reversed the promotive potential of migration and invasion induced by LEMD1 in PC cells (Figure 6g). To conclude, these findings indicated that LEMD1 promoted cell aggressiveness by the activation of the mTORC1 signaling pathway in PC.

## Discussion

As reported in recent decades, LEMD1 was upregulated in several cancers and played key roles in cancer progression. However, limited research has explored the expression, function and molecular mechanisms of LEMD1 in PC. In this study, we, for the first time, showed that LEMD1 was abnormally overexpressed in PC and was significantly related to PC prognosis. Upregulated LEMD1 could promote PC cell cycle progression and apoptosis resistance through p53 suppression and activation of the mTORC1 signaling, which, in turn, facilitates cancer cell proliferation and metastasis. Therefore, LEMD1 was recognized as a meaningful target for prognosis and therapy in PC.

Molecular signatures not only provide therapeutic biotargets but also could be identified to improve screening and early diagnosis in malignancies. However, almost most of the markers applied to predict, monitor and treat malignancies presented a lot of limitations due to the sensitivity, specificity and cost-effectiveness. Cancer-testis antigens (CTAs), a novel class of tumor antigens (TAAs) that are normally biased toward expression in the testis and are always induced in cancer cells, have been increasingly reported to be abnormally dysregulated in multiple cancers and possess the potential as tumor markers that can tremendously aid screening, prognostic factors, disease development and therapy [15,16]. Overexpression of CTAs has been correlated with advanced progression and poorer survival, highlighting a crucial role of CTAs in tumorigenesis [^16–18^]. With the update of CTAs, LEMD1 was first recognized and reported by Yuki D et al. in 2004 [5]. LEMD1 was shown abnormally upregulated in various kinds of malignancies, such as the oral squamous cell carcinoma, gastric cancer, colorectal cancer and prostate cancer. LEMD1 could function to be a meaningful marker for the prognosis and progression of such cancers [^5–8^]. In this study, evidenced from a series of public data (GEPIA, TCGA and GEO), we first showed that LEMD1 was highly expressed in PC and predicted poor survival. Of interest, all PC cell lines expressed a higher level of LEMD1. All meaningful findings indicated the oncogenic potential of LEMD1 and encouraged us to investigate its biological role in PC aggressiveness.

In recent decades, LEMD1 has been reported to facilitate cancer aggressiveness known as follows. For instance, Sasahira T et al reported that LEMD1 knockdown could inhibit the invasion, adhesion and migration of cancer, vascular or lymphatic vascular endothelial cells, suggesting the function of LEMD1 to be a novel tumor progressive factor and a potential marker for treatment and prognosis of oral squamous cell carcinoma [7]. In gastric cancer, upregulated LEMD1 contributes to cancer cell growth, apoptosis and cell cycle via the activation of PI3K/AKT signaling [6]. MiR-135b and host gene LEMD1 could facilitate the oncogenicity driven by nucleophosmin-anaplastic lymphoma kinase (NPM-ALK) and empower IL-17-producing immunophenotype, via the activation of signaling transducer and activator of transcription 3 (STAT3) [19]. Moreover, LEMD1 frequently played a key role in the maintenance of cancer-initiating cells (CICs)/cancer stem cells (CSCs) labeled with high capacity of initiation, self-renewal, differentiation and progression in colorectal cancer [20]. Herein, we explored the roles of LEMD1 by loss and gain in PC cells. By *in vitro* and *in vivo* experiments, we found that LEMD1 knockdown significantly inhibited, whereas ectopic LEMD1 promoted PC cell growth, migration and invasion. Of importance, by GSEA to extend findings, the LEMD1 level was closely related to the cell cycle and p53 signaling in PC samples, postulating the promotive influences of LEMD1 on cell proliferation and considering the cell cycle and apoptosis, which were generally proved to be powerfully responsible for the regulation of cell viability [10,21]. For cell cycle analysis, our results showed that LEMD1 knockdown arrested the cell cycle at G0/G1, whereas ectopic LEMD1 presented opposite trends. Mechanically, LEMD1 induced the negative regulation of p21 and p27, the well-known cyclin kinase inhibitors, which repressed the activation of CDK2 and CDK4 and negatively corelated with SKP2 in cancerous samples [22,23]. CDK2 and SKP2 expression was positively modulated by LEMD1 in PC cells; however, no significant differences were found for CDK4. All findings in this aspect were consistent with the correlation analysis of TCGA. For cell apoptosis, we found that upregulated LEMD1 suppressed cell apoptosis, mechanically by the negative regulation of Bax/Bcl-2.

P53 protein, maintaining genome stability and monitoring cellular genome damage [24], has been fully proved as a transcriptional regulator of Cyclin B1, MCM2, GADD5, p21 and p27 and to be commonly monitoring G2/M and G1 phases to repair DNA damage [25]. The tumor suppressor p53, a transcription factor, can regulate the target gene level (such as the p21, p27, Noxa, Bax, Perp and Puma), which is critical for cell senescence, apoptosis, growth inhibition and cell cycle arrest [25,26]. Due to the effects of LEMD1 on the PC cell cycle, apoptosis and GSEA of GEO, we detected p53. Our study supported the repressive roles of LEMD1 in p53 signaling by dephosphorylation of Ser-15 and Ser-46. All in all, LEMD1 was fully considered to be most likely to inhibit the activation of p53 signaling, then affecting the cell cycle and apoptosis and thereby stimulating PC cell proliferation.

Accumulating findings have disclosed that the mTORC1 signaling pathway, constitutively activated in almost all kinds of malignancies, is widely involved in cancer cell survival, growth, angiogenesis, migration, invasion and chemoresistance [27,28]. For LEMD1, multiple underlying oncogenic signaling pathways have been reported. For instance, Li et al. elucidated the promotive roles of LEMD1 in gastric cancer cell growth by the activation of PI3K/AKT signaling [6]. Xu et al. reported that LEMD1 facilitated thyroid cancer cell growth and metastasis via epithelial mesenchymal transition and activation of Wnt/β-catenin signaling [29]. In this study, due to GSEA, LEMD1 was proved as an upstream positive regulator of the mTORC1 signaling pathway by phosphorylation in PC cells. In addition, blockage of mTORC1 effectively abolished the stimulative influences of cell growth, migration and invasion induced by LEMD1. All the results in this aspect suggested that LEMD1 facilitated cancer cell aggressiveness via mTORC1 signaling in PC.

Although we reported LEMD1-dependent tumorigenesis in PC for the first time, there are still several limitations in our study. First, we knockdown/overexpress LEMD1 in only one cell line, not many cell lines to prove the data obtained. In addition, the precise mechanism (indirect/direct) of LEMD1 participating in the p53 and mTORC1 signaling remains unclear. Besides, whether any mechanic relationships underlying p53 and mTORC1 signaling that are responsible for cancer aggressiveness by LEMD1 existed in PC? For above limitations, further explorations are needed to fill gaps in the future.

## Conclusions

Our study first revealed that LEMD1 was upregulated in PC and predicted poor overall and disease-free survival. Overexpression of LEMD1 facilitated PC cell proliferation and metastasis, inducing cell cycle progression and apoptosis resistance, by repression of p53 and activation of the mTORC1 signaling pathway. This may provide a new potential target for PC prognosis and therapy.

## Data Availability

All data and material used or analyzed during the current study are available from the corresponding author on reasonable request.
